# Diagnostic challenge: bilateral infected lumbar facet cysts - a rare cause of acute lumbar spinal stenosis and back pain

**DOI:** 10.1186/1749-799X-5-14

**Published:** 2010-03-05

**Authors:** Brett A Freedman, Tuan L Bui, S Timothy Yoon

**Affiliations:** 1 Department of Orthopaedic Surgery, Emory University School of Medicine, Emory Spine Center, Altanta, GA 30329, USA

## Abstract

Symptomatic synovial lumbar facet cysts are a relatively rare cause of radiculopathy and spinal stenosis. This case and brief review of the literature, details a patient who presented with acutely symptomatic bilateral spontaneously infected synovial facet (L4/5) cysts. This report highlights diagnostic clues for identifying infection of a facet cyst.

## Introduction

Lumbar facet cysts are a less common but well documented cause of compressive radiculopathy and lumbar spinal stenosis, with approximately 500 total cases reported in the literature [1-5]. The lumbar facet is a synovial-lined zygoaphophyseal joint, comprising the articulation between the inferior and superior articulating processes of the spinal vertebrae. The facet joint, like synovial lined joints of the appendicular skeleton, are prone to cyst formation as a manifestation of osteoarthritis. To date, infection of a lumbar facet cyst has not been reported in the literature. This case illustrates the clinical findings and outcomes associated with bilateral infected lumbar facet cysts.

## Case Report

### History

Our patient is a 63 year old overweight gentleman who presented to the emergency room with a three day history of progressive low back pain and pain radiating down the right worse than left leg in an L5 distribution. He also noted an acute onset of drop foot. He rated his pain as 10 out of 10. He reported that he has had a history of intermittent back pain, but no prior leg symptoms. He has diabetes, which was marginally controlled (HgbA1C was 7.4), coronary artery disease and one week prior to presentation he completed an 8 week course of radiation therapy for prostate cancer.

### Physical Examination

On examination, he was in significant pain. He had bilateral lower extremity weakness. His motor strength testing revealed 4/5 left and right iliopsoas and 4+/5 left and right quadriceps, hamstrings and gastrocnemius muscles, all of which appeared to be pain induced reductions of strength. Additionally, he had 3/5 left and right tibialis anterior (TA) and extensor hallucis longus (EHL) function. His peroneals were also weak (4-/5). He had normal sensation to light touch and pin prick. His deep tendon reflexes were 2+ bilaterally. He had a normal upper extremity neurological and digital rectal exam.

### Imaging and Labs

**Figure 1:**
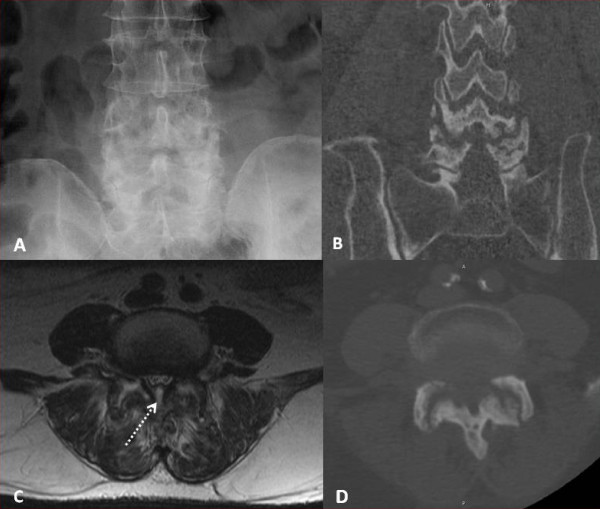
**Advanced degenerative changes of the L4/5 facets are seen on these AP radiograph and CT scan images**. (1A, B and D) Note the subchondral sclerosis and cystic changes. The axial T2 MRI image shows a focal fluid-like collection in bilateral L4/5 facet joints with contiguous extension into the midline dorsal epidural space (dotted line) (1C). Additionally, there is heterogeneous increased signal in the paravertebral muscles. There is no evidence of spondylodiscitis or paravertebral muscle abcess.

**Figure 2:**
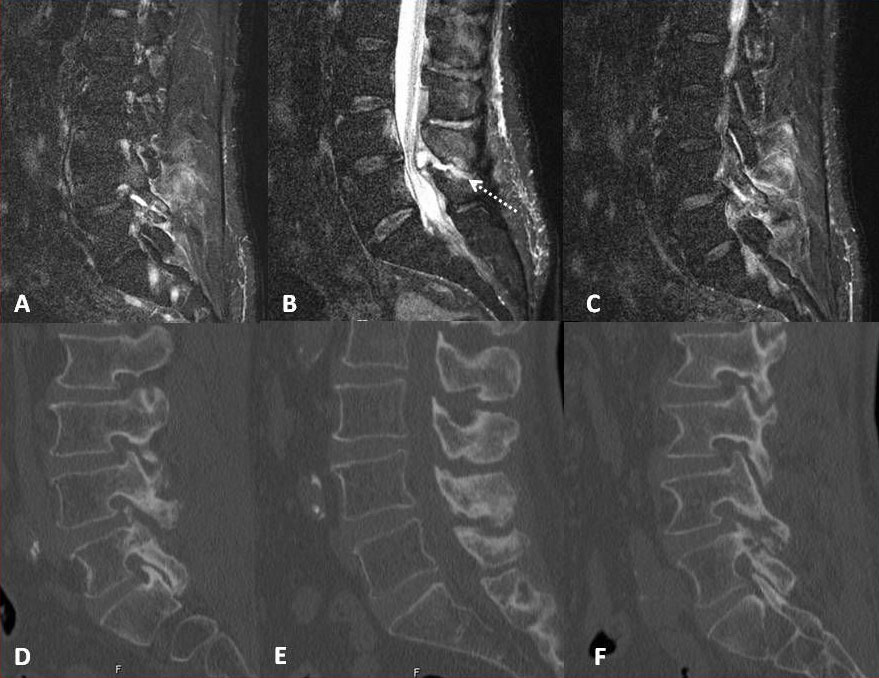
**Right to left sagittal T2 MRI images with their associated CT sagittal reconstructions beneath, show the fluid-like (isodense and isointense to CSF) signal in the right greater than left facet joints, as well as diffusely increased signal in the adjacent paravertebral muscles**. (2A and C) The midline sagittal MRI image shows the compressive epidural portion of the cyst, with a stalk that trails into the interspinous space (dotted line), where it communicates with the facet cysts. (2B) The CT images show non-specific chronic destructive changes to the L4/5 facet, typical of uncomplicated lumbar facet cysts. (2D, E and F)

Plain radiographs and a CT scan demonstrated severe arthrosis at the L4/5 facet joints. (Figure 1) MRI revealed what appeared to be large degenerative bilateral L4/5 facet cysts with extensions into the interspinous and epidural space, causing severe compression of the thecal sac. (Figure 1 and 2) There was paravertebral muscle heterogenous hyperintensity on fat-suppressed T2 images. He was afebrile and had normal white cell count and blood sugars. Due to the unusual acuity of symptom presentation, potential for immune compromise given his medical co-morbidities and subtle MRI findings suggestive of local inflammatory response in the paravertebral muscles, an ESR and CRP were obtained. They were both markedly elevated. (ESR 103 mm/hr; CRP 33.2 mg/dL) His admission and subsequent laboratory results are located in Table 1.

**Table 1:** Pertinent lab values.

	Emergency Department	POD#1	At Discharge	8 week follow-up
WBC (/mcL)	9,800	7,700	7,200	7,600

CRP (mg/dL)	33.2	19.6	16.5	.27 (0.033 at 3mo)

HgA1C (%)	7.4			

POD#1 = postoperative day one, WBC = white blood count (normal value < 11,100/mcL), CRP = C-reactive Protein (normal value < 0.8 mg/dL), HgA1C (Hemoglobin A1C, therapeutic goal < 6%).

### Operation and Pathological Findings

The clinical exam, imaging studies and laboratory findings all suggested this patient's symptoms were due to a L4/5 degenerative facet cyst causing symptomatic lumbar stenosis; however the markedly elevated CRP and ESR and inflammatory signal on MRI was worrisome for an infectious etiology. He was admitted to the hospital and taken to the OR the following day for a decompression and possible fusion of L4/5. Intraoperatively, exposing the L4/5 facets revealed voluminous cysts that expressed frank pus upon incision. As a result, we decided to stage this patient's surgeries. At the first stage we performed subtotal L4 and L5 laminectomies, near-total facet capsulectomy, partial facetectomy and thorough lavage to widely eradicate the infected structures. The infection appeared to be completely contained within the facet cysts. Tissue samples were sent for culture and pathology, which grew out Methicillin-Resistant Staphylococcus Aureus (MRSA). Pathology showed chronic and acute inflammatory changes without evidence of neoplasm. Infectious disease was consulted and he was started on intravenous Vancomycin, which was continued for a total of 6 weeks. Two days following the initial procedure, he underwent a direct lateral interbody fusion (X-LIF, NuVasive, Inc, San Diego, CA) with BMP-2 (InFuse, Medtronic, Inc, Minneapolis, MN) and posterior pedicle screw instrumentation.

### Postoperative Course

After his decompression surgery, the patient did well, with cessation of radicular symptoms, and was discharged on the 4^th ^postoperative day following the subsequent X-LIF. Upon discharge from the hospital, his pain level improved to 5/10, with pain solely in his back. His motor strength normalized in all groups except for EHL, TA and P, which only improved to 4/5. However, this was sufficient to eliminate his drop foot gait. At his 2 week clinic appointment, he was afebrile and his wound was healing without complication.

Unfortunately, our patient was a visitor to this country who permanently resided in the Virgin Islands. He had been here for his cancer treatments, but upon resolution of his back and leg symptoms, he returned to his home. He was scheduled to return to our clinic 2 weeks, 6 weeks, and 3 and 6 months postoperatively, but only came for a 2 and 8 week visit. At his 8 week visit, he had completed his IV antibiotic therapy two weeks prior and denied any pain. He rated pain in his legs and back as 0/10. He still had some slight L5 weakness (4+/5 EHL, TA) on examination; however, this did not affect his gait. His laboratory values had normalized. (Table 1) Due to his living situation, follow-up at 3 and 6 months was obtained telephonically and demonstrated no evidence of recurrent leg symptoms or infection.

## Discussion

The clinical presentation, management and outcomes of aseptic lumbar facet cysts have been reported [1-6]. In 2004, Epstein performed a comprehensive review of the 15 published case series, which provides defining characteristics of this pathology[2]. Facet cysts are detected in 0.6 - 10% of MRI scans of the lumbar spine [1-7]. The L4/5 level is most commonly affected and cysts most commonly occur in patients 60-65 years of age[1-3,5,8]. L5 radiculopathy is the most common primary complaint, although 95-100% of patients will have low back pain, as well[1,2,9].

Facet sagittal orientation (> 45 degrees) and facet arthrosis are present in over 77% of patients with symptomatic lumbar facet cysts [9-11]. As in the appendicular skeleton, the primary response of synovial joints to arthritis, is the over-production of synovial fluid, which in turn raises the intra-capsular pressure. In some patients, weak or thin areas in the facet capsule give way, the result is a focal mushroom-like swelling[1,12]. With time cystic fluid dehydrates, and the cyst itself undergoes myxoid degeneration. Classic cystic appearance on MRI (isodense and isointense with CSF) occurs in as few as 57% of facet cysts[9]. These space occupying lesions can compress nerve roots, causing radiculopathy (57-75%) or neurogenic claudication (25%)[2,9,13].

Although cases of successful nonoperative treatment have been reported with steroid injections or image-guided aspiration, for the most part, symptomatic synovial cysts require excision[1,2,9,14]. While agreement exists for surgical intervention of symptomatic facet cysts, the extent of surgery needed is debatable. Surgeons tend to favor either decompression alone or decompression and fusion, with decompression alone being the more commonly reported approach[1,5]. Concurrent spondylolisthesis, especially in the presence of significant low back pain, is the most common reason for adding an arthrodesis[1,3,5,12]. The lack of prospective cohort studies require surgeons to base their treatment plan on hypothesis and interpretation of case series which report good-excellent results (in > 75% of cases) for both approaches[1,5,12]. Those who advocate arthrodesis tend to point to two primary issues. First, the cyst is only an effect, the true cause is the underlying facet arthrosis and possibly instability[3,12]. Simply excising the cyst will not treat the cause. Conversely, the rate of recurrence following laminectomy alone appears to be quite low, averaging < 3% across published series[1,13]. Second, patients with lumbar facet cysts overwhelmingly have abnormal motion segments and low back pain[2,3,9,12]. Excision and decompression alone does not address these concomitant pathologies and may worsen segmental instability[3,9]. However, the rate of re-operation for symptomatic instability appears to be low as well (2% in Lyons et al. series of 194 patients)[5].

Our patient had chronic low back pain, sagittally oriented facets (> 45 degrees) with extensive cystic and sclerotic changes. We performed significant facetectomies and near-total capsulectomy to widely debride the infection; thus we elected to fuse his spine. His recent XRT exposure and his underlying marginally controlled diabetes made him vulnerable to infection--Class B host[15]. Hypertrophic synovium in facet cysts, devoid of a basement membrane, allowed MRSA to localize and develop into a closed space infection. This sequence of hematogenous seeding and subsequent infection is common to other synovial joints[16]. This case clearly demonstrates that this can occur in lumbar facet joints, as well. Further, debridement of the infected tissue, prolonged culture-specific antibiotic and stabilization through instrumented spinal fusion can successfully eradicate this rare form of infection and result in an excellent clinical outcome (= complete symptom resolution, no recurrence)[1,12].

This case report highlights diagnostic clues that suggest infection of an underlying facet cyst. The key findings appear to be rapid progression of symptoms, associated elevation in CRP and ESR and paravertebral muscles edema. Symptomatic neurological compression in uncomplicated facet cysts develops over time as degeneration progresses. Only 7% of cases present within 7 days of symptom onset, perhaps dues to intra-cystic hemorrhage[1,9,17]. In patients presenting with acutely progressive lumbar stenotic or radiculopathic symptoms which are attributed to lumbar facet cysts, the possibility of infection of the cysts should be considered and evaluated.

## Consent

Written informed consent to publish could not be obtained despite reasonable attempts. The patient cannot be identified from the case report and there is no reason to believe that they would object to its publication.

## Competing interests

The authors declare that they have no competing interests.

## Authors' contributions

All authors contributed in writing this case report, and have all read and approved the final manuscript.
